# Progress in Neurology

**Published:** 1903-02-28

**Authors:** 


### Progress in Neurology.

**Syphilis and Insanity.**—The various ways in which syphilis may cause insanity are defined by Mott[1] as follows :—(1) By moral shock. A psychopathic individual may become infected, and the moral shock may be so great as to lead to insomnia, and this to melancholia, mania, or delusional insanity. (2) By the effect of the poison and anæmia on the general nutrition of the body in the secondary stage, especially in a neuropathic or psychopathic individual. (3) Syphilis may produce meningitis, usually basal, gummatous tumours, endo-arteritis and periarteritis, especially within the first four years of infection. The complexus of symptoms is most variable, and often simulates general paralysis. The epileptiform fits, the progressive dementia accompanied by drowsy stupor, with intervals of restoration of intelligence, attacks of depression or excitement, with delusions of persecution, closely resemble the similar events of general paralysis. But some features are very different ; in particular the headache and tenderness of the head on percussion, which are never found in general paralysis. (4) Syphilis is a potent cause of arterio-sclerosis, which frequently causes brain softening, paralysis, and post hemiplegic dementia. (5) Syphilis is by far the most important factor, though not essential, in the production of general paralysis of the insane. This is borne out by many facts; in the first place, signs of syphilis are found in 55 per cent. of men dying from general paralysis, and in 25 per cent. of the women; secondly, a history of syphilis is found in 80 per cent. of cases admitted to asylums; thirdly, general paralytics cannot be inoculated with syphilis; and, lastly, in cases of juvenile general paralysis a history or evidence of congenital syphilis is almost invariable. In the discussion on this paper, Shuttleworth and Beach said that as far as could be ascertained syphilis played a very small part in the production of congenital idiocy, only about 2 per cent. presenting stigmata of that disease.

**Cerebral Compression.**—Cushing[2] has recently made a series of experimental investigations on this subject by trephining the skull of animals and exerting pressure by an indiarubber bag filled with mercury. He finds that when the pressure exerted is slight a distinct dilatation is produced in the pial veins of the exposed convolutions. As the pressure is increased the venous dilatation increases further, and the optic papilla also shows dilated veins. When the compression is still further increased, so that it equals or slightly surpasses the capillary blood-pressure, the convolutions become blanched whilst the veins remain dilated as before and are filled with dark blood. Such a condition of the cortical substance may account for the apathy or stupor which is associated with marked increase of intracranial pressure, such as may result from subdural hæmorrhage. This blanching of the cortex is shortly followed by evidences of stimulation of the vaso-motor centre, the carotid blood-pressure rises, and the capillary vessels are forced open and the circulation thus restored in the cortex—there is thus a compensatory mechanism which acts reflexly. As the pressure is increased the cardiac centre holds out longer than the respiratory, so that the heart keeps on beating after respiration ceases. The failure of the respiratory centre is preceded by an early stage of slow and shallow breathing, and a later one of Cheyne Stokes type. These experimental results thus coincide with what has been observed clinically on man, and afford valuable data for deciding on operative interference.

**Motor Aphasia from Injury.**—Newman[3] records three cases of motor aphasia from injury. In the first one the patient was thrown off a bicycle, and remained unconscious for six days; on the third day epileptiform fits began to occur, at first few in number but afterwards becoming very frequent. These fits began in the right hand and gradually spread over the whole body. As he recovered consciousness it was found that motor aphasia was present; and on the tenth day after the accident complete paralysis of the right arm and leg came on. On operation it was found that no fracture of the skull was present, but beneath the dura mater there was a quantity of dark fluid blood. Drainage tubes were inserted. Two days later the patient had recovered power in the right arm and leg, but it was ten days before the power of speech returned. Recovery was eventually complete. In the second case the skull was fractured; the patient was unconscious for four days, on the last of which twitchings began in the right side of the face and the right arm. The patient was trephined over the motor speech centre, and within a day or two the twitchings ceased, whilst the power of speech was recovered in about a fortnight.

**Puerperal Aphasia.**—Under this title Sinclair[4] includes cases of loss of speech during the later months of pregnancy, as well as in the strictly puerperal period. He records a case and gives an analysis of eighteen others. Some of these are certainly of neurotic origin, others are due to embolism, but there is a fair residuum where the blood vessels show no sign of degeneration, where there is no cardiac disease, no albuminuria, and no evidence of neurotic habit. The cause of these residual cases is probably thrombosis. This opinion is borne out by several considerations; in the first place, some of the cases present evidence of thrombosis occurring elsewhere in the body; and secondly, it is well known that there is a great increase in the amount of fibrin in the blood during the later months of pregnancy. A striking fact about these cases is that there is a large proportion of recoveries from primary attacks; the loss of speech may be only transitory, or recovery may take place in a few weeks or months. There is a great liability to recurrence of the aphasia in a succeeding pregnancy, and such a recurrent attack is likely to be much more lasting than the first one and to be accompanied by paralysis of the right arm and leg. It is, therefore, important to warn any such patient of the danger of again becoming pregnant, or if pregnancy does occur to terminate it before the fifth or sixth month.

**Hysterical Mutism.**—Lawrie[5] has recorded three cases of hysterical mutism, which had lasted one year, five years, and 33 years respectively. The condition, which occurs as frequently in men as in women, in contradistinction to hysterical aphonia, often follows a hysterical crisis, or after fright or shock. The complications are the various stigmata of hysteria, such as contracted fields of vision, hemi-anæsthesia, motor paralysis, and contractures. The diagnosis has to be made from ordinary motor aphasia, the mutism of insanity, and malingering. From the first-named condition it is distinguished by the active intelligence, the enhanced power of gesture language, and the rapidity with which the patient hastens to make known his thoughts in writing; whereas in organic motor aphasia the patient is very rarely altogether mute, but can still utter a sound of some sort—a syllable, a word, a phrase, or an emotional expression—and the power of writing is usually more or less interfered with. In the mutism of the insane delirium or mania co-exist; whilst in malingering the person overacts the part and he may be tricked or surprised into speaking. All the successful methods of treatment aim at restoring the normal relation between the attention and the speech centre, as by suggestion in either the hypnotic or the waking state, or by Faradism or cold douches. Isolation from friends is often essential.

[1] Brit. Med. Jour., Oct. 18, 1902. [2] Amer. Jour. Med. Sci., Sept. 1902. [3] Lancet. [4] Lancet, July 26. [5] Birm. Med. Jour., July.

(*To be Continued.*)

